# Novel intravesical therapeutics in the treatment of non-muscle invasive bladder cancer: Horizon scanning

**DOI:** 10.3389/fsurg.2022.912438

**Published:** 2022-07-26

**Authors:** Kelly Ward, Mark O Kitchen, Suresh-Jay Mathias, Farhat L Khanim, Richard T. Bryan

**Affiliations:** ^1^ The Bladder Cancer Research Centre, University of Birmingham, Birmingham, United Kingdom; ^2^ School of Medicine, Keele University, Stoke-on-Trent, United Kingdom; ^3^ New Cross Hospital, The Royal Wolverhampton NHS Trust, Wolverhampton, United Kingdom

**Keywords:** intravesical, non-muscle invasive bladder cancer, gemcitabine, immune checkpoint inhibitors, monoclonal antibodies, gene therapy, electromotive therapy, hydrogels

## Abstract

**Introduction:**

Non-muscle-invasive bladder cancer (NMIBC) is a common and heterogeneous disease; many patients develop recurrent or progress to muscle-invasive disease. Intravesical drug therapy is a pillar in the current management of NMIBC; notwithstanding, Mitomycin C (MMC) and Bacillus Calmette-Guérin (BCG) have numerous limitations including international supply issues, and local and systemic toxicity. Here we review novel intravesical therapeutic options and drug delivery devices with potential for clinical use in the treatment of NMIBC.

**Methods:**

PubMed, ClinicalTrials.gov and Cochrane Library searches were undertaken. Systematic reviews, meta-analyses, randomised controlled trials, single-arm clinical trials and national/international conference proceedings were included.

**Results:**

Novel intravesical drugs, including chemotherapeutic agents, immune checkpoint inhibitors, monoclonal antibodies and gene therapies, have demonstrated varying efficacy in the treatment of NMIBC. Current evidence for the majority of treatments is mostly limited to single-arm trials in patients with recurrent NMIBC. Various novel methods of drug delivery have also been investigated, with encouraging preliminary results supporting the intravesical delivery of hyperthermic MMC and MMC hydrogel formulations.

**Conclusions:**

Novel therapeutic agents and drug delivery systems will be important in the future intravesical management of NMIBC. As our understanding of the molecular diversity of NMIBC develops, molecular subtyping will become fundamental in the personalisation of intravesical treatments. Further randomised studies are urgently required to investigate the efficacy of novel intravesical treatments and novel regimens, in comparison to current standards-of-care, particularly in the context of international BCG shortages.

## Introduction

**Figure 1:**
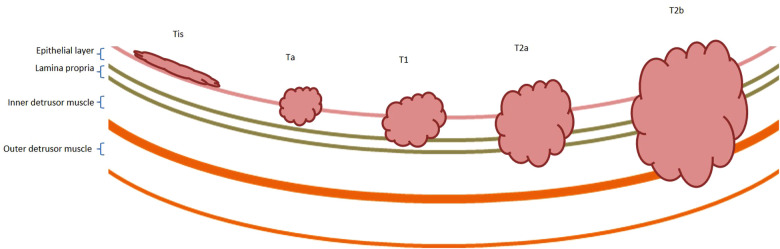
**Non-muscle and muscle-invasive bladder cancer.** Bladder cancer is described as non-muscle or muscle invasive. Non-muscle invasive bladder cancer includes Tis, Ta and T1 disease, as described by the TNM staging system (131). Tis lesions are typically located on the luminal surface. Ta tumours extend into the urothelium, and T1 tumours extend into the lamina propria. Muscle-invasive bladder cancers include T2, T3 and T4 disease. T2 tumours extend into the inner half (T2a) and outer half (T2b) of the detrusor muscle, respectively. T3 tumours extend in to the perivesical fat and lymph nodes and T4 tumours invade organ systems. T3 and T4 tumours are not shown within this diagram.

Bladder cancer (BCa) is the twelfth most common cancer worldwide (1), but as a consequence of an international lack of research funding, treatment has made very little progression over the last 25 years, with both pharmacological and surgical treatment options remaining largely unchanged (2). Most patients (75%–80%) present with “early-stage,” non-muscle-invasive disease (NMIBC: stages Tis/Ta/T1, Figure 1) (3); but many are sub optimally managed with recurrences occurring in up to 80%. Furthermore, 40%–50% of cases progress to muscle-invasive Bca (MIBC: stages T2+, Figure 1) which carries a 5-year survival rate of only 27%–50% (4–7), emphasising the need for early diagnosis and appropriate primary treatment.

NMIBCs are highly heterogeneous both clinically and biologically (8). High-grade NMIBCs have a high mutation burden, multiple copy number changes and loss of tumour suppressors (*TP53*, *RB1*) more akin to MIBC, whereas “low-grade NMIBCs exhibit oncogene activation (*FGFR3*, *RAS*) in a relatively normal genome (9). Epigenetic changes, including histone tail modifications, microRNA expression and DNA hypermethylation, also have an association with NMIBC progression and phenotype (10, 11). Recently, “subtyping” based on gene expression has offered further insights into NMIBC biology (12), yet risk stratification and treatment selection remains entirely based upon clinico-pathological characteristics, without the inclusion of biomolecular information (3). Notwithstanding, the majority of NMIBCs are considered amenable to bladder preservation with transurethral resection of bladder tumour (TURBT) and adjuvant intravesical therapy, albeit accompanied by adverse effects (AEs) (13, 14).

The European Association of Urology (EAU) guidelines stratify NMIBC into low-, intermediate-, high- and very high-risk disease, which indicates the recommended adjuvant intravesical therapy or the need for upfront radical cystectomy (RC) (3). It is widely accepted that in low-risk tumours, single dose, post-TURBT mitomycin C (MMC) reduces the risk of recurrence and can be considered curative. Intermediate-risk disease should be managed with six instillations of MMC or one year of Bacillus Calmette-Guérin (BCG), and high-risk disease by BCG induction, followed by up to three years of maintenance BCG therapy. Patients with very high-risk disease should be offered immediate RC, or full-dose BCG for one to three years**.** Patients typically undergo radiological, cystoscopic, and sometimes urinary biomarker surveillance, depending upon their risk category (3). Given the intensive surveillance schedules, potentially prolonged treatment courses, and high recurrence rate (up to 85%), NMIBC is considered one of the most expensive malignancies to treat (15).

MMC and BCG both have unpleasant local side effects including chemical cystitis, and haematuria (14). Intravesical BCG can also lead to severe systemic side effects including flu-like symptoms, pneumonia and sepsis, and has ongoing global production and supply issues (14, 16–18). Despite adequate treatment, some NMIBC becomes resistant to MMC and/or BCG (19, 20). BCG-unresponsive and relapsing NMIBC is particularly challenging to treat, with few management options other than radical cystectomy (RC). Therefore, novel and effective, chemotherapeutic agents are urgently needed for the treatment of NMIBC (21).

Here we present a narrative review which explores the evidence for the use of novel intravesical drug treatments and regimens in the treatment of NMIBC, either alone or in-combination with existing standards-of-care. We will consider all new intravesical drugs and intravesical drug delivery methods which have been tested in a clinical trial setting, but which are not yet clinically approved for the treatment of NMIBC by national or international guidelines.

## Methods

PubMed, Clinicaltrials.gov and the Cochrane Library were interrogated with the search terms: “non-muscle invasive bladder cancer”, “superficial bladder cancer”, “transitional cell carcinoma”, “urothelial cell carcinoma”, “intravesical therapy” and “intravesical drug delivery”. Meta-analyses, systematic reviews, randomised controlled trials (RCTs) and single-arm clinical trials were included from 2000 to 2022. Internationally and nationally-recognised conference proceedings detailing preliminary results were included for ongoing and unpublished clinical trials. Due to the small number of research in this field, studies of novel intravesical therapeutics in newly diagnosed NMIBC (stages Tis/Ta/T1), recurrent disease, and BCG “refractory”, “relapsing” and “unresponsive” disease were included. We excluded pre-clinical studies, and papers which were not published in the English language.

In this review, we use the terms “refractory”, “relapsing” and “unresponsive” NMIBC, per the definitions in the European Association of Urology (EAU) guidelines (3). BCG-refractory tumours are carcinoma *in situ* (Tis) or high-grade NMIBCs which remain after three months of BCG re-induction or maintenance therapy. BCG-relapsing tumours occur after completion of maintenance BCG, despite an initial treatment response. BCG-unresponsive tumours are BCG-refractory tumours and recurrences of high-grade tumours or Tis within 6 or 12 months of adequate BCG therapy, respectively. All AEs discussed are classified by the National Cancer Institute's Common Toxicity Criteria for Adverse Events (22).

## Results

A summary of novel intravesical drugs and drug delivery methods for the treatment of NMIBC is shown in Table 1.

**Table 1:** Summary of novel intravesical drugs and drug delivery methods.

Novel intravesical drug or delivery method	Mechanism of action	Studies	Clinical trial quality	Patient outcomes
**Novel intravesical drugs**				
ALT-803 with BCG	Interleukin-15 “superagonist”	Chamie et al., 2021.	Phase 2/3	59% CR at 12 months predicted (*n* = 80)
Apaziquone	Bio-reductive alkylating agent	Karsh et al., 2018.	RCT	39% RR at 24 months (*n* = 1614)
BC-819	Recombinant DNA plasmid which expresses diphtheria toxin A	Gofrit et al., 2014.	Phase 2	33% CR at 3 months (*n* = 39)
CG0070	Oncolytic adenovirus expressing GM-CSF	Packiam et al., 2018.	Phase 2	30% CR at 12 months (*n* = 45)
Docetaxel	Stabilises microtubule assembly and inhibits mitosis	Barlow et al., 2013.	Phase 1/2	40% RFS at 12 months∼25% RFS at 36 months (*n* = 54)
Docetaxel and Gemcitabine	As above and a pyramidine nucleoside antimetabolite	Steinberg et al., 2022.	Phase 2	42% RFS at 24 months (*n* = 93)
Durvalumab	Programmed cell death ligand-1 monoclonal antibody and immune checkpoint inhibitor	Pending.	Phase 2	Trial Ongoing
Gemcitabine	Pyramidine nucleoside antimetabolite	Addeo et al., 2010.	Phase 3	72% RFS at 36 months (*n* = 54)
Gemcitabine and Everolimus	Pyramidine nucleoside antimetabolite and mTOR inhibitor	Dalbagni et al., 2017.	Phase 1/2	16% RFS at 12 months (*n* = 19)
Nadofaragene firadenovec	Recombinant adenovirus vector containing the interferon α-2b gene	Boorjian et al., 2021.	Phase 3	31% RFS at 12 months (*n* = 151)
Pembrolizumab	Programmed cell death protein 1 monoclonal antibody and immune checkpoint inhibitor.	Pending.	Phase 1/2	Trial ongoing
Valrubicin	Anthracycline topoisomerase inhibitor	Steinberg at al., 2000.	Phase 2	21% CR at 30 months (*n* = 90)
Vicinium	Recombinant fusion protein to epithelial cell adhesion molecule	Shore et al., 2020.	Phase 3	50–52% RFS at 12 months (*n* = 127)
**Novel intravesical delivery methods**				
Chemohyperthermia	Heat energy encourages urothelial MMC uptake	Zhao et al., 2021	Meta-analysis	29.5% at 24 months (*n* = 156)
Electromotive Drug Administration	MMC absorption enhanced through iontophoresis, electrophoresis, electroporation	Tan et al., 2019.	RCT	53% RFS at 3 months (*n* = 33)
Gemcitabine-releasing Intravesical System Device	Semipermeable silicone tube delivering sustained gemcitabine release	Pending.	Phase 1	Pending outcome (*n* = 12)
Nanoparticle albumin bound paclitaxel	Albumin enhances drug transportation across the cell membrane.	McKiernan et al., 2014.	Phase 2	36% RFS at 12 months (*n* = 28)
UGN-102 Hydrogel	Increases MMC retention time	Chevli et al., 2022.	Phase 2	61% CR at 12 months (*n* = 63)

The table above shows a summary of novel intravesical drugs and drug delivery methods which have been tested in clinical trials for the treatment of NMIBC. The type of clinical study and the study outcomes are shown. CR, Complete response; RFS, Recurrence Free Survival.

### Novel intravesical drugs

#### Gemcitabine

**Figure 2:**
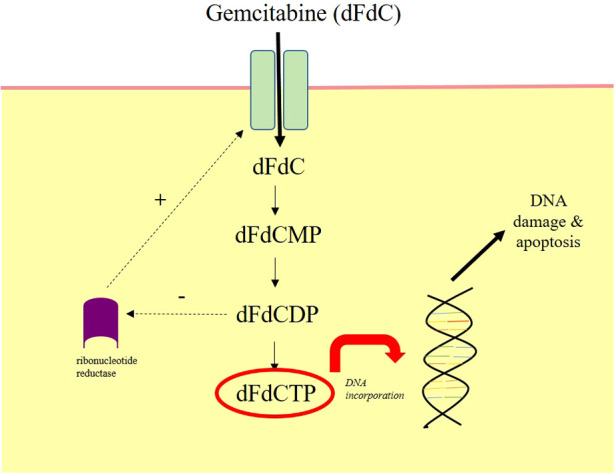
**Mechanism of action of intravesical gemcitabine.** Gemcitabine (dFdC) is prodrug. It moves intracellularly following intravesical administration through nucleoside transporters. It is phosphorylated by kinases into gemcitabine monophosphate (dFdCMP), gemcitabine diphosphate (dFdCDP) and finally gemcitabine triphosphate (dFdCTP). dFdCTP incorporates into the DNA strand and inhibits DNA synthesis, which mediates cellular apoptosis. dFdCDP also inhibits ribonucleotide reductase, which drives further dFdC uptake. Diagram redrawn from Ueno et al, 2007 (24).

Gemcitabine (2′-deoxy-2′,2′-difluorocytidine-hydrochloride, beta isomer) is a pyramidine nucleoside antimetabolite. It was originally investigated for its anti-viral properties, but was incidentally found to have potent anticancer effects. Following cellular uptake, gemcitabine is converted into the active metabolite difluorocytidine-hydrochloride-5-diphsoph-5-triphosphate, which terminates DNA replication and initiates apoptosis (23, 24) (Figure 2). As a parenteral chemotherapeutic agent, it is currently licensed for metastatic BCa (25) and is commonly used in combination with cisplatin for the neoadjuvant treatment of MIBC (5).

Given its efficacy in metastatic BCa, there have been several studies investigating intravescial gemcitabine in the management of NMIBC. Typical dosing is 1000–2000 mg in 50–100 ml of saline, aiming to produce urinary concentrations of approximately 40 mg/ml (26). Marker lesion studies use cystoscopy to assess BCa size and morphology pre- and post-treatment, and suggest that intravesical gemcitabine is most effective when delivered as a course; bi-weekly intravesical administration (over three weeks) has demonstrated a complete response (CR, defined as complete disappearance of the lesion at nine weeks) in 40% of patients (27). Intravesical gemcitabine is generally well-tolerated, and is associated with a low frequency of grade 2 and 3 AEs such as mild dysuria, cystitis, and haematuria (28–30).

Intravesical gemcitabine may be more effective than MMC in the treatment of NMIBC (28). In a meta-analysis of 335 NMIBC patients from five RCTs, recurrence rates were significantly lower in patients receiving an eight week course of gemcitabine form to a six week course of MMC (OR 0.44, 95% CI, 0.24–0.78). Gemcitabine was also associated with a significantly lower rate of AEs than MMC.

This meta-analysis included the results of five clinical trials, but there is significant study heterogeneity, including in drug dosing and delivery schedules (28). Furthermore, many of the studies included did not carry out double blinding and the available studies may have been prone to publication bias. Further large-scale RCTs are required to compare the treatment efficacy of intravesical gemcitabine to MMC.

Intravesical gemcitabine may also be superior to MMC in the treatment of recurrent NMIBC. In 2010, a phase III trial randomised 109 patients with recurrent NMIBC to weekly, intravesical treatment with a six week course of gemcitabine (2000 mg) or a four week course of MMC (40 mg). After 36 months, recurrence free survival (RFS) was significantly greater in the gemcitabine group (72% vs. 61% vs., *p* = 0.002), with fewer AEs associated with intravesical gemcitabine than MMC (29, 30)

The efficacy of intravesical gemcitabine in comparison to BCG remains unclear. In one randomised controlled trial (RCT), 64 patients with high-risk NMIBC received weekly intravesical gemcitabine (2000 mg, over six weeks) or BCG (5 × 10^8^ colony forming units) (31). After 44 months, the recurrence rate was significantly higher in the gemcitabine group (53% vs. 28% *p* = 0.037), suggesting that gemcitabine is less effective than BCG in high-risk tumours. Conversely, a study of 80 high-risk NMIBC patients (who had failed treatment with one course of BCG) were provided with an intravesical course of gemcitabine (2000 mg) or a further course of BCG (81 mg). Both drug regimens consisted of a six week induction period followed by maintenance doses at three, six and twelve months. After one year, 53% of patients receiving gemcitabine developed recurrent disease compared to 88% of those receiving BCG (*p* = 0.002) (30). Intravesical gemcitabine monotherapy therefore shows promise in the management of NMIBC, including high-risk disease.

Intravesical gemcitabine has also been investigated as a combination therapeutic in early clinical trials, including with everolimus, an mTOR inhibitor which enhances the cytotoxic effect of gemcitabine (32, 33). Bi-weekly, intravesical gemcitabine (2000 mg) was administered for six weeks, and daily oral everolimus (10 mg) for 12 months to 19 patients with BCG-refractory NMIBC (32). Only 16% of patients were disease free at one year, but 53% suffered grade 3 or 4 AEs, leading to early termination of the study. Other trials are ongoing, and are evaluating gemcitabine in combination with intravesical BCG (34), docetaxel (35), cabazitaxel, and cisplatin (36).

#### Docetaxel

Docetaxel is a chemotherapeutic agent which stabilises microtubule assembly, inhibits mitosis, and initiates apoptosis (37). It is in clinical use for metastatic breast and prostate cancers, and has been shown to inhibit *in vitro* and *in vivo* growth in BCa models (38, 39). In 2013, a phase I/II trial evaluated six weeks of intravesical docetaxel followed by twelve monthly maintenance doses (5–75 mg) in 54 patients with recurrent NMIBC who had failed to respond to standard intravesical therapy with BCG, MMC or interferon. 59% had an initial response, classified as a negative biopsy and urine cytology six weeks after intravesical treatment. RFS was 40% at one year, but dropped to 25% after three years (40). Overall, docetaxel was well-tolerated and associated with very few grade 1 and 2 AEs (41).

Docetaxel has also been investigated in combination with intravesical gemcitabine (35). A case-series investigated a six week course of weekly intravesical gemcitabine (1 g) and docetaxel (37.5 mg) in 45 patients with recurrent NMIBC (following previous BCG treatment) or a contraindication to BCG treatment. Overall, this study demonstrated a RFS of 54% at 12 months (35). A recent retrospective study has also been published which compares the efficacy of intravesical gemcitabine and docetaxel therapy to intravesical BCG and interferon α-2b in 290 NMIBC patients. The 2-year high-grade RFS was 42% for the gemcitabine and docetaxel group compared to 51% for the BCG and interferon α-2b group (42).

#### Apaziquone

Apaziquone is a bio-reductive alkylating agent and a derivative of MMC. It is a pro-drug which is activated by intracellular reductases to generate cytotoxic species, which initiate DNA damage and apoptosis (43). Apaziquone has demonstrated favourable efficacy in BCa models *in vitro*; in some cases with superiority over MMC (44).

Clinically beneficial effects have also been demonstrated using intravesical apaziquone in NMIBC (45, 46): 46 patients with Ta/T1 NMIBC underwent TURBT followed by six weekly doses of intravesical apaziquone (4 mg). A CR was seen in 67%, defined as a histological response (46). Despite these encouraging findings, subsequent trials have failed to replicate or confirm the efficacy of apaziquone.

A phase III RCT allocated 1614 NMIBC patients to treatment with TURBT plus a single intravesical dose of apaziquone (4 mg/40 ml) or TURBT plus an intravesical saline placebo. The treatment arm only saw a 7% reduction in the two-year recurrence rate of NMIBC (which was non-significant) (47). Possible reasons for lack of drug efficacy included haematuria (post TURBT) which may increase metabolism of apaziquone. Notwithstanding, some evidence from a pooled analysis found that delaying intravesical instillation of apaziquone (more than 30 minutes post TURBT) significantly improved the two-year recurrence rate compared to the placebo (35% vs 47%, *p* = 0.0014) (47, 48).

Most alkylating agents are associated with severe myelosupression (49); however, 40 mg of intravesical apaziquone is undetectable in the systemic circulation post administration, and is therefore safe and well tolerated (45, 50). For example, AEs are mild and similar to those seen with intravesical placebo treatments, including dysuria, bladder spasm and urgency (47). Given the encouraging early data and favourable safety and tolerability profiles, further clinical trial evaluation of intravesical apaziquone is warranted.

#### Immune checkpoint inhibitors

Immune checkpoints are inhibitory protein complexes which downregulate immune responses and prevent autoimmune cell death. Immune checkpoints are frequently dysregulated by many cancer types; this confers protection from host immune destruction and promotes cell survival, and ultimately tumour progression and metastatic spread. Immune checkpoint complexes consist of a cell-expressed ligand and a receptor (51); monoclonal antibody blockade of this ligand-receptor interaction is a current focus of investigation in BCa therapeutics.

Of particular importance is programmed cell death protein 1 (PD-1), an immune checkpoint inhibitor which is activated by programmed cell death ligand-1 (PD-L1) that supresses T-cell responses (52). PD-L1 is highly expressed in a significant proportion of NMIBCs and can be associated with poor prognosis (53, 54). **Pembrolizumab** is a monoclonal antibody against PD-1, its intravenous use was approved in 2017 for locally-advanced and metastatic urothelial carcinoma (55). Early clinical studies evaluating intravesical pembrolizumab for the treatment of NMIBC are now also ongoing (56, 57).

**Durvalumab** is also a monoclonal antibody targeted to PD-L1; it was previously approved in the U.S for the treatment of metastatic BCa, but later withdrawn due to limited clinical efficacy (58–60). However, early-stage clinical trials are currently investigating intravesical durvalumab for NMIBC (61).

#### Vicinium

Vicinium is a recombinant fusion protein of a single-chain variable monoclonal antibody fragment to the epithelial cell adhesion molecule (EpCAM), and a truncated fragment of *pseudomonas* exotoxin A (62). The monoclonal antibody fragment localises the drug to EpCAM-positive BCa cells, where the *pseudomonas* exotoxin A blocks protein synthesis, and mediates cellular apoptosis (63–65).

Clinical trials have demonstrated that intravesical vicinium may be effective against BCG-relapsing NMIBC. 126 patients received an induction course of six weeks of bi-weekly intravesical vicinium followed by six-weeks of weekly instillations (30 mg). Maintenance therapy consisted of further fortnightly intravesical instillations (66, 67). 40% of patients with Tis and 71% of patients with Ta/T1 disease remained disease free at three months (67). In a similar trial of 22 patients treated with six-week course of weekly vicinium (up to 30 mg), 41% had CR at 3 months (68). In 2021, the U.S Food and Drug Administration denied the approval of intravesical vicinium for the treatment of NMIBC (69).

Vicinium is selective for EpCAM-positive BCa cells; with such a targeted mechanism of action, AEs should be limited (67). Also, due to its small molecular weight, almost all vicinium remains within the bladder following intravesical administration (68, 70), hence AEs tend to be localised and mild (grade 1–2); nevertheless, AEs have been reported in up to 52% of patients (67, 71).

#### Valrubicin

Valrubicin, an anthracycline topoisomerase inhibitor and derivative of doxorubicin, is the only intravesical therapy approved in the U.S for the treatment of BCG-refractory NMIBC in patients unfit for cystectomy. The recommended regimen is 800 mg per week, for six weeks (72). The exact mechanism of valrubicin is incompletely understood; however, it is thought to involve interference with nucleic acid metabolism and initiation of cell cycle arrest (73).

In a pivotal trial, 90 patients with recurrent NMIBC (who had received at least one course of BCG) were treated with six weekly 800 mg instillations of intravesical valrubicin; 21% had a CR with a median follow-up time of 30 months. The median time to treatment failure was almost 18 months (74). Further clinical studies of intravesical valrubicin have found similar treatment response rates (73, 75–78).

High rates of AEs have been reported with intravesical valrubicin, with up to 86% of patients developing local reactions including urinary frequency, dysuria and skin irritation (78). AEs tended to occur immediately after drug administration, although only 5% of patients discontinue treatment due to side effects (73). Systemic AEs are uncommon, with a handful of reports of azotaemia and renal failure (76, 78)

#### ALT-803

Intravesical BCG immunotherapy is routinely given in high-risk NMIBC, but many patients develop recurrent disease. To improve efficacy, a combination therapy of BCG with intravesical ALT-803 has been proposed (79). ALT-803 is a recombinant complex of an interleukin-15 (IL-15) “superagonist”, and a human IL-15 receptor *α*-sushi domain/human IgG1 Fc fusion protein (IL-15 alpha Se/Fc fusion protein). IL-15 activates Natural Killer and CD8+ T cells and IL-15 alpha Se/Fc fusion protein enhances its biological activity. In a phase I clinical trial, a six-week course of weekly intravesical ALT-803 (escalating doses) and BCG (50 mg) was delivered to nine patients with BCG-naïve NMIBC. Intravesical treatment was delivered following TURBT in patients with evidence of Ta/T1 disease. All patients had a CR at 24 months, which was defined as a normal cystoscopy and negative biopsy or voided urinary cytology (79). In a case-report, a 91-year-old male with recurrent NMIBC, who was unsuitable for cystectomy, was treated with six doses of weekly intravesical ALT-803 (400 µg) and BCG (50 mg). The patient remained disease-free for more than 19 months (80). In a recent phase II/III trial, intravesical ALT-803 was delivered in combination with BCG to 80 patients with NMIBC Tis with or without additional Ta/T1 disease. Preliminary results show a 72% complete response rate (CRR) which was defined as absence of Tis at any time point. It is predicted that the CRR at 12 months will be 59% (81).

A large-scale RCT is currently ongoing and evaluating the efficacy of intravesical ALT-803 and BCG combination therapy compared to BCG monotherapy, in patients with BCG-naïve NMIBC (82). A clinical trial was planned to assess the efficacy of ALT-803 monotherapy, but was terminated in 2021 due to the coronavirus pandemic (83). AEs have unfortunately been reported in all patients receiving combination intravesical ALT-803 and BCG, and include hypertension, haematuria and urinary tract infection (84, 85). It is unclear whether BCG or ALT-803 or their combination, was the principal contributor to these side effects.

#### Gene therapy

Gene therapy uses viral or plasmid vectors to introduce exogenous DNA into a host cell to produce a therapeutic response. It can be used as an oncological treatment, for example, where the introduction of genes may activate an immune antitumour response (86, 87).

**Nadofaragene firadenovec** (rAd-IFNα/Syn3) is a recombinant adenovirus vector which contains the human recombinant interferon α-2b gene and Syn3. IFN α-2b is an immune modulator, which has been shown to be safe as an intravesical therapy (88), but has limited therapeutic efficacy, potentially due to its short half-life (89, 90). To overcome this, rAd-IFNα/Syn3 has been developed to introduce the IFN α-2b gene into urothelial cells, which sustains intravesical IFN α-2b release (91). Syn3 is a polyamide surfactant which enhances gene transfer into BCa cells, and appears to be key for IFN α-2b gene expression (91). Intravesical rAd-IFNα/Syn3 has shown promising results in the management of BCG-unresponsive NMIBC. In a phase III clinical trial, 151 patients were treated with three, 12 weekly intravesical doses of rAd-IFNα/Syn3 (75 ml). 60% of patients had a CR at three months and 31% of patients were free from a high-grade recurrence at 12 months (90). 66% patients reported mild AEs; however, these were generally acceptable and led to only three patients stopping treatment (90). Overall, rAd-IFNα/Syn3 appears a promising intravesical therapy candidate, and further studies are currently ongoing to evaluate long-term efficacy (92).

**CG0070** is an oncolytic adenovirus which selectively and preferentially replicates in cancerous cells with retinoblastoma-pathway (Rb-pathway) defects. It encodes and expresses the granulocyte-macrophage-colony stimulating factor (GM-CSF): a cytokine which stimulates granulocyte production and initiates antitumour immune responses (93). Rb-pathway defects are common in BCa and are associated with recurrence, disease progression, and high mortality rates (94).

To improve vector transduction across the BCa cell membrane, CG0070 is routinely delivered with the surfactant dodecylmaltoside (95). In a 2018 phase II clinical trial, a six week course of weekly intravesical dodecylmaltoside 0.1% (75 ml) and CG0070 (1X10^12^ VP) was delivered to 45 patients with treatment resistant NMIBC. All patients had failed treatment with two previous courses of intravesical therapeutics, with at least one course of BCG treatment. Six months post-treatment with CG0070, 47% of patients had a CR and only 2% of patients had progressed to muscle-invasive disease (96). In a 12 month follow-up analysis of 57 patients, 30% had a CR (97). Reported AEs included immunological symptoms such as fever and chills (97).

A six-week combination therapy of intravesical CG0070 and IV pembrolizumab (400 mg) is now also being investigated for patients with BCG-unresponsive NMIBC (98, 99).

**BC-819** is a recombinant DNA plasmid containing a regulatory H19 gene promoter that drives BCa cell expression of the diphtheria toxin A which in-turn inhibits local protein synthesis and mediates cell death (100, 101). When delivered to 39 patients with recurrent NMIBC, a six-week course of intravesical BC-819 (20 mg) led to a three month CRR of 33% (102). Unfortunately, the follow-up trial was terminated due to lack of drug efficacy (103).

### Novel intravesical drug delivery methods & devices

**Figure 3:**
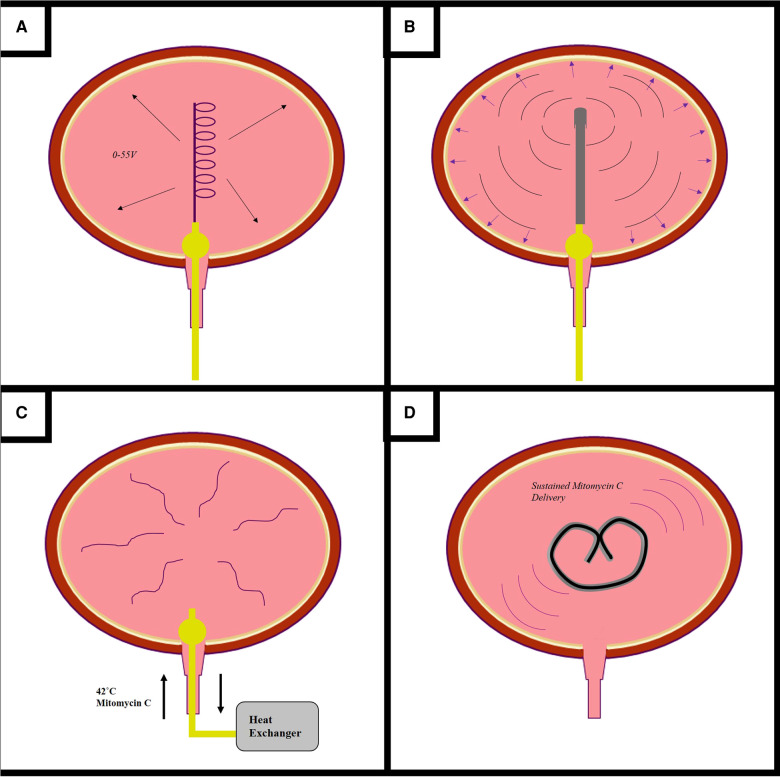
**Novel devices for the administration of intravesical drugs.** Novels drug delivery methods are being developed to enhance the efficacy of mitomycin c (MMC) and other intravesical therapeutics (**A**–**D**). Electromotive drug delivery (**A**) consists of inserting a cathode into into the bladder and applying an electrical current to enhance urothelial drug uptake. The Synergo system (**B**) uses a radiofrequency antenna to heat the bladder wall. The Combat BRS system (**C**) externally heats MMC, for intravesical circulation *via* a three-way catheter. The GemRIS device (**D**) is a silicone tube which provides sustained intravescial drug delivery over a two week period.

Intravesical therapy requires direct drug delivery into the bladder through a urinary catheter. Following drug instillation, patients are required to maintain continence for one to two hours to ensure adequate urothelial exposure. Variations in bladder volume and rate of urine production lead to variability in drug concentration. Furthermore, early-voiding can limit drug efficacy (104). Some intravesical drugs, such as BCG, require repeat bladder instillations, which increases the risk of urinary tract infections and is highly inconvenient for both patients and clinicians (3). Various drug delivery devices and techniques have been designed to enhance and sustain the delivery of intravesical therapeutics in the treatment of NMIBC **(**Figure 3**)**.

#### Electromotive drug administration & chemohyperthermia

**Electromotive drug administration** and **chemohyperthermia** are well-described techniques which may enhance intravesical drug efficacy in the treatment of NMIBC. Trial evidence has been reported and reviewed elsewhere in the literature, and is outside the remit of our review (105, 106). Electromotive drug administration (EMDA) is a device-assisted therapy which uses an electrical current to enhance delivery and urothelial absorption of MMC through a combination of iontophoresis, electrophoresis, and electroporation. One electrode is inserted into the bladder *via* a spiral catheter, another placed on the skin of the lower abdomen, and an electrical current of 0–30 mA DC at 0–55 V is passed between them (107). EDMA is not currently recommended in the treatment of NMIBC, due to insufficient clinical trial evidence (105, 108).

**Chemohyperthermia** (CHT) uses heat energy to increase the urothelial uptake of MMC; it may also enhance BCa cell death through hypoxic mechanisms and by enhancing antitumour immunity (107). There are two main types of CHT. Firstly, the Synergo system uses radiofrequency microwave energy to directly heat the bladder wall. A catheter containing an integrated radiofrequency antenna is inserted into the bladder and heats the epithelium to 42°C (109). The Synergo system is well-explored within the literature, and current EAU guidelines recommend that patients with BCG-unresponsive NMIBC who are unfit for RC may be considered for chemohyperthermia using the Synergo device (3, 110).

The Combat BRS device also uses heat energy. MMC is externally heated to 43˚C and then instilled and recirculated through the bladder using through a three-way catheter. The Combat BRS device is easy and cheap to use, but its use is less explored within the literature (109).

#### Gemcitabine-releasing intravesical system device

The **gemcitabine-releasing intravesical system** (GemRIS) device was originally designed for the sustained delivery of lidocaine in the treatment of interstitial cystitis; however, it is under evaluation for gemcitabine delivery in the setting of NMIBC (111, 112). The device consists of a semipermeable silicone tube which is inserted into the bladder *via* a urinary catheter, from which gemcitabine is released over a two-week period (111). An early-stage clinical trial evaluated the safety of GemRIS in 12 patients with NMIBC (112). One group received the device for two seven-day periods and the second group received the device for two 21-day periods. Full trial data is pending publication (113).

#### Nanotechnology

Nanocarriers are materials between 1–200 nm, which have been developed to transport drugs. They are becoming increasingly common in clinical medicine, and various nanocarriers are currently approved in the treatment of ovarian, haematological and gastrointestinal malignancies (114).

**Nanoparticle albumin bound paclitaxel** (nab-paclitaxel) consists of the chemotherapeutic drug paclitaxel and the delivery component albumin. Paclitaxel (a taxane drug) targets the cytoskeletal protein tubulin and prevents microtubule disassembly and mitosis. The albumin component enhances drug transport across the cell membrane into the intracellular cytoplasm. Nab-paclitaxel is less toxic than unbound paclitaxel and can be administered in higher doses (115). In 2014, 28 patients with recurrent NMIBC received a six-week course of weekly intravesical nab-paclitaxel (500 mg). 36% of patients had an initial CR, and 67% were progression-free at an average follow up period of 21 months (116). On later analysis of this patient cohort, the one year and three year RFS rates were 32% and 18%, respectively (117).

**Nanoparticle albumin bound rapamycin** (nab-rapamycin) has also been explored as a novel intravesical therapeutic. Rapamycin is an inhibitor of the mTOR signalling pathway, which is thought to be implicated in the progression of NMIBC to MIBC (118). Early-stage clinical trials have found that intravesical ABI-009 (1000 mg) is safe and tolerable. Further trial data are awaiting publication (119).

#### Hydrogels

Hydrogels, particularly those based upon non-synthetic materials that can dissolve, provide sustained intravesical drug release, and may improve drug efficacy. There are two types of intravesical hydrogels: **mucoadhesive substances** which use chemical or physical interactions to anchor onto bladder mucosa, and **floating platform hydrogels** which generate microbubbles and float within the bladder*. In vivo* BCa models have demonstrated that hydrogels can deliver and release a wide-range of chemotherapeutic agents for BCa including MMC, doxorubicin and epirubicin (120).

Phase II clinical trials have shown that intravesical MMC therapy may be curative in some patients with low-risk NMIBC. In 82 patients with a visible recurrence, 37% of patients treated with a four week course of standard intravescial MMC (without surgical intervention) had a CR at three months (121).

**Figure 4:**
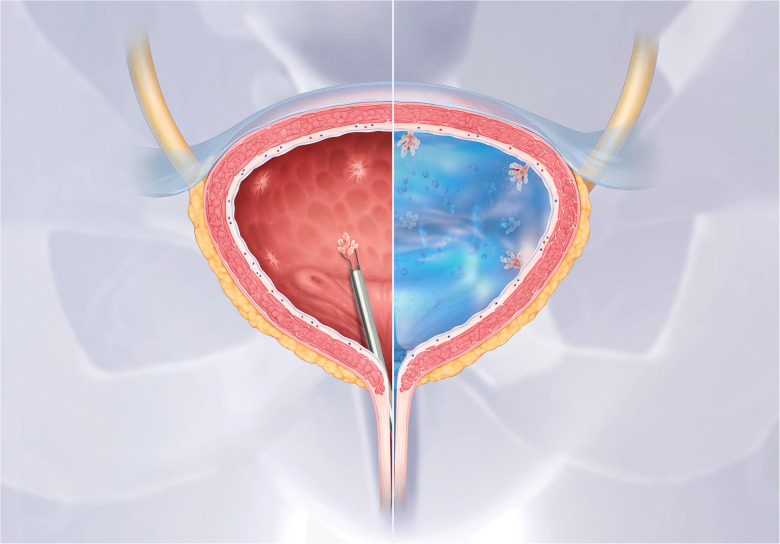
**UGN-102 hydrogel for intravesical mitomycin C instillation.** Figure supplied by UroGen. UGN-102 is a mitomycin hydrogel. When instilled into the bladder UGN-102 turns from a liquid into a gel substance, which increases drug retention time for up to six hours.

**UGN-102** is a MMC hydrogel which may increase bladder retention time and improve MMC's efficacy as a primary chemoablative therapy. UGN-102 is administered in a liquid state and turns into a semi-solid gel at body temperature Figure 4). The gel breaks down over a six-hour period and is eliminated through urination. In a single-arm trial, 63 patients with low-grade, intermediate-risk NMIBC were treated with six, weekly instillations of UGN-102. At 12 months, 65% of patients had a CR (122). To further investigate UGN-102 a RCT is currently ongoing to compare a 6-week course of UGN-102 to TURBT only in patients with low-grade, intermediate-risk NMIBC (123).

## Discussion

Despite advances in understanding of the natural history and molecular mechanisms of NMIBC development, intravesical MMC and BCG remain the predominant intravesical drugs (14). BCG-unresponsive and relapsing NMIBC is particularly challenging to treat, with few management options other than RC. RC is unsuitable for many patients with MMC or BCG “failure”, due to the inherent comorbidities and frailty of age typically occurring in these patients. RC is associated with reduced quality of life, potentially significant post-operative physical and psychological detriment, and is considered “over-treatment” for many patients (124). Novel and effective chemotherapeutic or immunological agents are therefore urgently needed for the treatment of NMIBC, particularly for patients whose tumours persist or progress despite MMC or BCG therapy.

Intravesical gemcitabine appears particularly promising, has demonstrated greater efficacy than MMC in several trials, and has an acceptable safety and toxicity profile (28, 29). Pending ongoing clinical trials and completed trial data analyses, it is expected that gemcitabine may become routinely available in clinical practice for the management of intermediate-risk NMIBC. Results of early-stage clinical trials are also promising for “targeted” therapies including immune checkpoint inhibitors and monoclonal antibodies. The selectivity of these drugs should minimise their toxicity and side-effects (52–54, 56, 59, 68). Chemohyperthermia and hydrogels are promising and may enhance the clinical efficacy of intravesical MMC delivery (107, 122); with international BCG supply issues, improving MMC efficacy may be particularly important for patients with intermediate- and high-risk NMIBC (125).

Many of the reviewed clinical studies are of single-arm design and some are retrospective case series; therefore, robust comparisons between novel therapeutics or the current standard-of-care with MMC or BCG, cannot currently be made. Furthermore, as outcome assessment is un-blinded, most of these studies may be prone to bias. Drug response and NMIBC recurrence is routinely assessed by cystoscopy and/or urine cytology. As Tis and small papillary tumours may be challenging to identify (due to user dependence of flexible cystoscopy and poor sensitivity of urine cytology for low-grade non-exfoliative tumours), recurrences may have been missed on follow-up. Considering variations in follow-up protocols and methods of surveillance, clinical outcome data from many of these trials described should be interpreted with caution.

Following single-arm, phase II clinical trials, both intravesical valrubicin and IV pembrolizumab were granted fast-track approval in the U.S for the treatment of BCG-refractory, and BCG-unresponsive NMIBC, respectively (74, 126). The approval of valrubicin has been criticised, as follow-up studies have demonstrated a CRR of less than 20% at three months (73, 75–78). Various novel therapeutics have demonstrated greater efficacy than valrubicin, with rAd-IFN*α*/Syn3 leading to a CRR of >50% at three months (90). Thus, early-approval should also be considered for other more effective intravesical therapies.

Combination therapies are common in the treatment of most cancers, and should be considered for the intravesical treatment of NMIBC. Combining two or more anticancer agents can provide a synergistic effect, prevent drug resistance and improve patient survival. This approach could also reduce the therapeutic doses of each drug required, potentially minimising drug toxicity (127). Indeed, several centres are currently trialling a combination approach to the intravesical treatment of NMIBC, some examples include gemcitabine and BCG, and pembrolizumab and BCG (34, 128).

NMIBC is a heterogeneous disease encompassing a spectrum of genomic, pathological and clinical phenotypes. Developments in technology for genomic analyses have identified molecular subtypes of NMIBC, potentially permitting the future stratification of BCa treatments and the subsequent delivery of personalised intravesical therapeutic approaches in the management of NMIBC (12). Although such precision medicine in NMIBC has not yet been realised, several drugs that target and “reset” genome-wide epigenetic modifications are being investigated in pre-clinical studies, and have shown extremely promising results in pre-clinical BCa models (129, 130).

## Conclusions

Novel intravesical therapeutics are urgently needed for the treatment of NMIBC, particularly during the current BCG crisis. Gemcitabine and chemohyperthermia-assisted MMC have both demonstrated superiority over standard MMC therapy in some types of NMIBC. Early-stage clinical trials have also shown very promising results for immune checkpoint inhibitors, monoclonal antibody therapies, and gene therapies. Unfortunately, hitherto, novel intravesical therapeutics have most often been assessed within single-arm study settings, and therefore high quality RCTs are required to drive changes in clinical practice. In the near future, it is hoped that tumour (NMIBC) genomic profiling will allow more accurate risk stratification and targeted intravesical treatments.
